# Magnetic Resonance Imaging in Axial Spondyloarthritis: Beyond the Sacroiliac Joints—Focus on Facet Joint Involvement

**DOI:** 10.3390/diagnostics16193148

**Published:** 2026-09-28

**Authors:** Martin Mitev, Lyubomir Sapundzhiev, Konstantin Batalov, Tanya Sapundzhieva, Zguro Batalov

**Affiliations:** 1 Department of Internal Diseases, Medical Faculty, Medical University of Plovdiv, 4002 Plovdiv, Bulgaria; k.batalovv@gmail.com (K.B.); taniasapundjieva@abv.bg (T.S.); zzbatalov@gmail.com (Z.B.); 2 Department of Rheumatology, University Hospital Pulmed, 4002 Plovdiv, Bulgaria; sapoundjiev@abv.bg; 3 Clinic of Rheumatology, University Hospital Sofiamed, 1113 Sofia, Bulgaria; 4 Clinic of Rheumatology, University Hospital Kaspela, 4000 Plovdiv, Bulgaria

**Keywords:** magnetic resonance imaging (MRI), axial spondyloarthritis, facet joints, sacroiliac joints, inflammation

## Abstract

Axial spondyloarthritis (axSpA) is a chronic inflammatory disease of the sacroiliac joints (SIJs) and spine, encompassing non-radiographic axSpA (nr-axSpA) and radiographic axSpA (r-axSpA) or ankylosing spondylitis (AS). Early diagnosis is challenging, particularly in nr-axSpA, due to the absence of radiographic sacroiliitis and overlap with nonspecific back pain. Imaging plays a pivotal role in disease classification and monitoring. To synthesize current evidence on inflammatory and structural facet joint involvement in axSpA, with particular emphasis on MRI characterization, available scoring approaches, differentiation from degenerative abnormalities, and the current limitations of facet joint assessment as a potential imaging biomarker. A review of current evidence regarding imaging modalities in axSpA, including conventional radiography, computed tomography (CT), MRI, radionuclide techniques, and musculoskeletal ultrasound (US), was performed. Particular emphasis was placed on ASAS/OMERACT MRI definitions and facet joint assessment. MRI enables early detection of inflammatory lesions such as bone marrow edema (BME), spondylitis, and enthesitis before structural damage becomes visible on radiographs. It also identifies structural changes including fat metaplasia, erosions, syndesmophytes, and ankylosis. Increasing evidence supports the clinical relevance of facet and costovertebral joint inflammation, which correlates with impaired spinal mobility and functional disability. However, inflammatory facet joint changes remain underreported in routine practice despite the development of semiquantitative scoring systems. Ultrasound complements MRI in assessing enthesitis but is limited in evaluating deep axial structures. MRI has become indispensable in axSpA diagnosis and monitoring. Standardized imaging definitions and structured reporting are essential to improve clinical integration and patient outcomes.

## 1. Introduction

Axial spondyloarthritis (axSpA) is a chronic, immune-mediated inflammatory disease predominantly affecting the axial skeleton, particularly the sacroiliac joints (SIJs) and the spine. The disease spectrum includes non-radiographic axial spondyloarthritis (nr-axSpA) and radiographic axial spondyloarthritis, traditionally referred to as ankylosing spondylitis (AS). Clinically, axSpA is characterized by inflammatory back pain, morning stiffness, progressive limitation of spinal mobility, and a potential decline in functional capacity over time [1,2]. Beyond axial manifestations, extra-articular features such as uveitis, inflammatory bowel disease (IBD), and psoriasis may occur, further complicating disease recognition and management.

Early diagnosis of axSpA remains challenging. Patients with nr-axSpA often present with typical inflammatory symptoms but lack definitive radiographic sacroiliitis according to the modified New York criteria [2]. Given that chronic back pain is highly prevalent in the general population, distinguishing inflammatory from mechanical (degenerative and traumatic) etiologies is frequently difficult. Consequently, diagnosis relies on a combination of clinical features, laboratory markers such as elevated C-reactive protein (CRP) or erythrocyte sedimentation rate (ESR), genetic testing for HLA-B27, and increasingly, advanced imaging modalities—particularly magnetic resonance imaging (MRI) of the sacroiliac joints [2]. At the same time, the increasing sensitivity of modern imaging introduces the opposite challenge of potential overdiagnosis. Imaging abnormalities suggestive of inflammation may also occur in healthy individuals and in association with mechanical stress, degeneration, or other non-inflammatory conditions. Accurate interpretation is therefore essential not only to reduce diagnostic delay but also to avoid disease misclassification, unnecessary investigations, and potentially inappropriate treatment.

This article is presented as a narrative review. A targeted, non-systematic search of the PubMed database was conducted for articles published from January 1985 through 31 August 2026. Search terms included combinations of ‘axial spondyloarthritis,’ ‘ankylosing spondylitis,’ ‘facet joint,’ ‘zygapophyseal joint,’ ‘magnetic resonance imaging,’ ‘MRI,’ ‘sacroiliac joint,’ ‘spinal inflammation,’ ‘facet joint inflammation,’ ‘facet joint ankylosis,’ and ‘scoring system.’ Original studies, consensus and recommendation documents, and relevant narrative, systematic, and scoping reviews addressing imaging findings in axSpA, with particular emphasis on spinal and facet joint involvement, were considered. Reference lists of relevant publications were also reviewed to identify additional studies of interest. Given the narrative design of this review, no formal systematic-review protocol, risk-of-bias assessment, or meta-analysis was performed. Although a previous scoping review by Acosta et al. systematically summarized grading systems for inflammatory changes of the lumbar facet joints on MRI, the present narrative review addresses a different and broader question: the relevance of facet joint involvement within the imaging spectrum of axial spondyloarthritis. Rather than focusing primarily on methodological comparison of lumbar facet grading systems, we synthesize evidence regarding inflammatory and structural facet joint abnormalities in axSpA, their associations with disease activity, spinal mobility, and structural progression, and the challenges involved in distinguishing inflammatory lesions from degenerative or mechanically related abnormalities. We additionally examine current MRI definitions and scoring approaches and identify the methodological gaps—including limited validation, heterogeneous acquisition and scoring methods, and insufficient evidence in non-radiographic axSpA—that currently restrict the diagnostic and clinical application of facet joint assessment. Accordingly, the aim of this review is to place facet joint imaging within the broader clinical and imaging context of axSpA and to define priorities for future standardization and prospective validation.

## 2. Imaging Modalities in Axial Spondyloarthritis

The introduction of the Assessment of SpondyloArthritis International Society (ASAS) classification criteria significantly broadened the conceptual framework of axSpA. Standardized definitions of spinal MRI lesions in axSpA have evolved over time. The 2022 ASAS MRI Working Group update refined definitions of inflammatory and structural spinal lesions and extended standardized assessment across multiple anatomical locations, including vertebral corners and endplates, facet joints, transverse processes, and other posterior elements [3]. These definitions provide a framework for consistent lesion characterization but should not be interpreted as a stand-alone diagnostic definition of axSpA. Unlike the modified New York criteria, which focus primarily on structural changes visible on conventional radiography, the ASAS criteria incorporate active inflammatory lesions detected by MRI and HLA-B27 positivity, enabling earlier classification of patients without established radiographic damage [4]. However, studies have demonstrated that the correlation between clinical symptoms and imaging findings may be inconsistent. A cross-sectional study of 1020 patients with persistent low back pain revealed that although more than half exhibited at least one spondyloarthritis feature, only 21% fulfilled the ASAS definition for sacroiliitis, highlighting the complexity of interpretation and potential diagnostic uncertainty [5].

Imaging therefore plays a central role in both diagnosis and disease monitoring of patients with axSpA. Major rheumatology organizations, including EULAR and the ACR, differ in their approaches to assessing axial spondyloarthritis (axSpA), particularly spinal involvement. EULAR recommendations place strong emphasis on the role of imaging modalities, especially magnetic resonance imaging (MRI) and conventional radiography, for the detection of both active inflammatory lesions (vertebral corner inflammation, erosions, fat metaplasia) and chronic structural changes (syndesmophyte formation, ankylosis) in the spine and sacroiliac joints, as well as for monitoring structural progression over time using standardized imaging definitions and scoring systems [6]. In contrast, the 2019 ACR/SAA/SPARTAN recommendations are primarily oriented toward clinical assessment of disease activity and therapeutic decision-making, with MRI recommended selectively when disease activity is unclear and imaging findings are expected to influence management, rather than for routine imaging assessment or scheduled monitoring of structural spinal progression [7].

Conventional radiography of the sacroiliac joints has historically been recommended as the initial imaging modality in suspected axSpA because of its accessibility, low cost, and ability to demonstrate established structural sacroiliitis. However, its limited sensitivity for early inflammatory disease has increasingly shifted attention toward MRI. Revised EULAR imaging recommendations presented at the 2026 EULAR Congress proposed a greater role for sacroiliac-joint MRI as an initial imaging modality in suspected axSpA. As these recommendations were newly presented at the time of the present review, their publication status and subsequent implementation should be considered when interpreting current imaging pathways [8]. Typical radiographic findings in the SIJs include erosions, subchondral sclerosis, pseudo-widening of the joint space, bony bridging, and complete ankylosis. In the spine, structural changes may involve vertebral corner erosions, vertebral squaring, syndesmophytes, disc calcification, and intervertebral ankylosis [9]. However, interpretation of SIJ radiographs is often challenging and associated with significant inter- and intra-observer variability, particularly in early disease stages [10]. This variability partly reflects the complex, irregular three-dimensional anatomy of the SIJs that is compressed into a two-dimensional image on plain radiographs, making subtle abnormalities difficult to interpret. In early disease stages, structural changes such as mild erosions, minimal sclerosis, or slight joint space alterations are often very subtle and may overlap with normal anatomical variants, age-related degeneration, or mechanical changes. Moreover, plain radiographs are relatively insensitive to early inflammatory changes, as they only detect structural damage rather than active inflammation. The grading of sacroiliitis is also partly subjective, particularly in borderline cases, which further contributes to disagreement between observers and even inconsistency by the same reader at different times.

Computed tomography (CT) can detect structural SIJ abnormalities more sensitively than conventional radiography [11]. Low-dose CT protocols have further enhanced visualization of syndesmophytes throughout the spine, especially in the thoracic region, which is frequently affected in axSpA [12]. CT has also revealed structural signs of sacroiliitis in a subset of patients undergoing abdominal imaging for inflammatory bowel disease, suggesting a potential overlap between gastrointestinal and musculoskeletal inflammation [12]. Despite these advantages, CT primarily detects structural changes rather than active inflammation and exposes patients to ionizing radiation, limiting its routine use for early diagnosis or longitudinal monitoring. Computed tomography is not included as an independent imaging modality in the imaging arm of the 2009 ASAS classification criteria for axial spondyloarthritis. In these criteria, sacroiliitis on imaging is defined by either definite radiographic sacroiliitis according to the modified New York criteria or active sacroiliitis on MRI. CT nevertheless has an important role in the sensitive detection and characterization of structural sacroiliac joint lesions, particularly erosions, sclerosis, and ankylosis [4]. More recently, revised ASAS–SPARTAN classification criteria for axSpA were developed and validated within the Classification of Axial SpA Inception Cohort (CLASSIC) study and presented at the 2025 American College of Rheumatology (ACR) Convergence, with subsequent presentation and discussion at the 2026 EULAR Congress. The revised framework places greater emphasis on imaging and incorporates a more focused set of clinical variables than the 2009 criteria. However, at the time of the present literature review, these revised criteria had been presented in congress proceedings and should therefore be distinguished from fully published peer-reviewed classification recommendations. Accordingly, when describing the established classification framework, this review refers to the 2009 ASAS criteria while acknowledging the emerging revised ASAS–SPARTAN criteria [13].

Magnetic resonance imaging (MRI) has fundamentally changed the diagnostic approach to axSpA by enabling detection of active inflammatory lesions before radiographic damage becomes apparent [9]. MRI uniquely visualizes bone marrow edema (BME) or osteitis, which represent key markers of active inflammation. According to the ASAS/OMERACT consensus definition, a positive MRI for active sacroiliitis requires the clear presence of bone marrow edema (BME)/osteitis in the subchondral bone, with an appearance that is highly suggestive of SpA [14]. However, the absence of a strict quantitative threshold remains a limitation of this definition, and careful consideration of lesion location and extent is necessary to avoid false-positive interpretations [15]. In addition to inflammatory lesions, MRI identifies structural changes including erosions, fat metaplasia, backfill, syndesmophytes, and ankylosis. Fat metaplasia and backfill, detectable on T1-weighted sequences, are particularly relevant as they reflect prior inflammation and are associated with subsequent radiographic progression [9]. For diagnostic evaluation of the SIJs, the ASAS–SPARTAN consensus recommends a minimum four-sequence MRI protocol comprising three semicoronal sequences—T1-weighted imaging for structural damage and fat-related changes, T2-weighted fat-suppressed imaging for active inflammation, and an erosion-sensitive sequence optimized for the bone–cartilage interface—together with a semiaxial T2-weighted fat-suppressed sequence for assessment of active inflammation [16]. MRI findings in the SIJs have been shown to correlate with histological and clinical features of axSpA [9]. Accordingly, in patients with suspected early disease in whom the diagnosis cannot be established on the basis of clinical and radiographic findings alone, MRI of the SIJs is strongly recommended. However, recent population-based studies have raised concerns regarding the specificity of sacroiliac joint bone marrow edema (BME) as an isolated MRI finding for axial spondyloarthritis. In a recent German cross-sectional MRI study including more than 11,000 individuals from the general population, sacroiliac BME was detected in approximately 30% of participants, a prevalence substantially exceeding that of reported axial spondyloarthritis. The presence of BME was associated with female sex, previous pregnancy, increased body mass index, physically demanding occupational work, and, in men, intensive recreational physical activity, demonstrating that SIJ BME is not specific to inflammatory disease. Furthermore, the study emphasized that MRI-detected BME, when assessed without adequate clinical correlation, may have limited diagnostic specificity. These observations underline the importance of interpreting MRI findings in conjunction with lesion morphology, anatomical localization, structural abnormalities, and the overall clinical context in order to minimize the risk of overdiagnosis and inappropriate therapeutic decisions. Collectively, these data support a more cautious and clinically integrated interpretation of MRI positivity within the ASAS classification framework [17]. Against this background, increasing attention has been directed toward imaging findings beyond isolated sacroiliac bone marrow edema, including inflammatory and structural abnormalities of the facet joints and other posterior spinal elements [3]. Facet joint involvement represents a recognized manifestation of axial disease; however, its incremental diagnostic value in patients with suspected axSpA remains uncertain [3,18]. In particular, facet joint effusion, periarticular bone marrow edema, and soft-tissue edema are not specific to axSpA and may also occur in degenerative or mechanically stressed joints [18]. Therefore, although systematic assessment of the facet joints may provide complementary information regarding the distribution and burden of spinal involvement, current evidence does not establish that these findings independently improve diagnostic specificity, resolve equivocal sacroiliac joint MRI findings, or reduce misclassification. Prospective studies including appropriate mechanical and degenerative control groups are required to determine their incremental diagnostic value [18].

Bone scintigraphy has also been investigated for the assessment of sacroiliac inflammation in early axial SpA, although its diagnostic performance varies according to the scintigraphic method used [19]. However, in 2008, Song et al. determined an overall sensitivity of about 50% and specificity not higher than about 80% for the diagnosis of sacroiliitis using bone scintigraphy [20]. Quantitative assessment using sacroiliac joint-to-sacrum uptake indices may improve the specificity of bone scintigraphy, although this may occur at the expense of sensitivity [21]. Unilateral sacroiliitis on scintigraphy also demonstrates relatively high specificity but low sensitivity, limiting its diagnostic utility in axSpA [22].

Although sacroiliac joint inflammation has traditionally been considered the hallmark of axial spondyloarthritis (axSpA), increasing evidence indicates that facet (zygapophyseal) joints represent an important and clinically relevant component of axial involvement. Facet joints are the only true synovial joints of the spine and, as such, are susceptible to inflammatory processes similar to those observed in peripheral synovial joints in spondyloarthritis (SpA). Six distinct inflammatory lesion types have been described in the spine: anterior and posterior spondylitis, spondylodiscitis, arthritis of the costovertebral joints, arthritis of the zygapophyseal (facet) joints, and enthesitis of spinal ligaments [23]. Structural changes include fatty infiltration, erosions, syndesmophyte formation, and ankylosis [23]. Spinal MRI is considered positive for inflammation when anterior or posterior spondylitis is identified in three or more vertebral sites, based on expert consensus [23]. Early vertebral corner inflammation, commonly referred to as a Romanus lesion, is characterized on MRI by low T1-weighted signal and high fluid-sensitive signal at the vertebral corners, reflecting active inflammatory change [24]. MRI has been shown to be more sensitive than conventional radiography in detecting such early inflammatory changes [24]. In more advanced disease, destructive discovertebral lesions, including Andersson lesions, may occur. These lesions can mimic infectious spondylodiscitis; however, MRI findings such as the absence of intradiscal or perivertebral fluid collections and relative preservation of intervertebral disc signal may favor a noninfectious origin [25]. Thus, MRI of both the SIJs and the spine allows simultaneous assessment of inflammation and structural damage, making it an essential tool not only for early diagnosis but also for monitoring disease activity and progression and evaluating treatment response.

Inflammatory facet-joint changes in axSpA include synovitis, joint effusion, and bone marrow edema; with disease progression, these abnormalities may ultimately culminate in ankylosis. Despite their potential clinical relevance, these changes are frequently underrecognized in routine imaging interpretation. A retrospective review of lumbar spine MRI reports in patients with chronic low back pain demonstrated low sensitivity (6–22%) for reporting inflammatory facet joint findings, with positive predictive values ranging from 25% to 100%; inflammatory features were most frequently identified at L4–L5 [26]. These findings suggest that inflammatory facet joint lesions may be substantially underreported in everyday clinical practice. Longitudinal data further support the clinical significance of facet joint involvement. In the Groningen Leeuwarden Ankylosing Spondylitis (GLAS) cohort, 25% of patients with ankylosing spondylitis (AS) exhibited partial or complete cervical facet joint ankylosis at baseline, and 13% developed new ankylosis over four years despite treatment with TNF-α inhibitors [27]. Facet-joint damage and progression may also occur without concomitant vertebral-body involvement. Facet-joint damage was associated with longer disease duration, a history of inflammatory bowel disease, uveitis or psoriasis, higher disease activity, and impaired spinal mobility, whereas facet-joint progression was associated with greater occiput-to-wall distance and more facet-joint damage at baseline [27].

Incorporating facet-joint assessment into radiographic scoring systems may provide additional value. The addition of the de Vlam cervical facet joint score to the modified Stoke Ankylosing Spondylitis Spine Score (mSASSS) resulted in the Combined AS Spine Score (CASSS), which identified more patients with definite structural damage and radiographic progression compared with mSASSS alone, while maintaining excellent interobserver reliability [28]. Moreover, cervical rotation correlated more strongly with CASSS than with mSASSS, underscoring its functional relevance [28]. An MRI study demonstrated that inflammatory lesions of the lumbar facet and costovertebral joints were associated with impaired spinal mobility and worse functional status, as reflected by BASMI and BASFI [29]. Together, these findings link facet-joint inflammation with restricted spinal mobility and functional impairment, supporting the potential clinical relevance of posterior spinal involvement in axSpA. Beyond axSpA, facet joint involvement has been described in other inflammatory rheumatic diseases. Facet-joint involvement is common in radiographic axSpA and may occur throughout the spine, with thoracic involvement reported frequently [30]. CT studies further demonstrate structural facet-joint abnormalities in axSpA. Ankylosis is particularly evident in radiographic axSpA, while syndesmophytes and erosions have been reported almost exclusively in radiographic disease [31]. Predominant involvement of the cervical facet joints has been described in PsA; however, the prevalence and clinical significance of facet-joint involvement in PsA remain insufficiently characterized [30]. Although rheumatoid arthritis has traditionally been considered to spare the lumbar facet joints, available evidence is limited. In a CT-based cross-sectional study, the prevalence of lumbar facet-joint changes did not differ significantly between patients with RA and age- and sex-matched controls [32]. Importantly, inflammatory facet-joint disease must be distinguished from degenerative facet arthropathy and other causes of facet-mediated pain. Facet-joint pain has been estimated to account for approximately 15–41% of chronic low back pain, although reported prevalence varies according to the clinical setting, diagnostic definition, and diagnostic procedure used [33]. In axSpA, MRI-detected facet-joint inflammation has been associated with restricted spinal mobility and functional impairment [29]. Although degenerative changes predominate in many patients, MRI-detected inflammatory facet-joint lesions—particularly bone marrow edema—may have distinct clinical relevance in axSpA, with facet-joint inflammation associated with restricted spinal mobility and functional impairment [29], and MRI facet-joint inflammatory scores associated with systemic inflammatory markers, including ESR and CRP [34]. Taken together, the available evidence supports facet-joint inflammation as a clinically relevant component of axial disease in axSpA. Facet-joint involvement has been associated with structural progression, reduced spinal mobility, and functional impairment. These observations warrant further investigation of facet-joint involvement across the axial disease spectrum. However, its incremental diagnostic and prognostic value beyond established clinical and imaging parameters remains uncertain and requires prospective validation.

## 3. MRI Assessment of Facet Joint Involvement

Facet joint abnormalities should be considered within the broader spectrum of spinal MRI lesions in axial spondyloarthritis. The updated definitions of spinal MRI lesions developed by the ASAS MRI Working Group include inflammatory and structural abnormalities involving posterior spinal elements, including the facet joints. These definitions distinguish inflammatory bone marrow lesions from structural abnormalities such as fat lesions, erosions, bone formation, and ankylosis and provide a standardized framework for anatomical localization and lesion description. These definitions are intended to improve consistency in MRI assessment and should not be interpreted as establishing the independent diagnostic specificity of facet joint abnormalities for axSpA [3].

MRI assessment of the facet joints requires evaluation of both active inflammatory and structural abnormalities. Fluid-sensitive fat-suppressed sequences, including short tau inversion recovery (STIR) or fat-suppressed T2-weighted imaging, are useful for identifying bone marrow edema and periarticular soft-tissue edema, whereas T1-weighted imaging facilitates assessment of structural abnormalities and marrow changes. Because facet joints are relatively small and obliquely oriented structures, lesion detection may be influenced by image quality, anatomical plane, slice thickness, and reader experience. At present, however, no universally accepted facet-specific MRI acquisition protocol for axSpA has been established, and published studies have used heterogeneous imaging approaches. This methodological variability limits direct comparison between studies and represents an important area for future standardization.

The growing recognition of facet joint (FJ) involvement in axial spondyloarthritis (axSpA) has stimulated the development of structured MRI-based scoring systems aimed at standardizing assessment and improving reproducibility. Historically, MRI evaluation of the spine in axSpA focused predominantly on vertebral corner inflammation and structural changes, while inflammatory lesions of the zygapophyseal (facet) joints were often overlooked. Evidence linking facet-joint inflammation to impaired spinal mobility and functional status [29], as well as to systemic inflammatory markers [34], has supported the development of more systematic MRI assessment approaches.

Early MRI studies of inflammatory facet arthropathy emphasized the detection of synovitis and joint effusion using fat-saturated sequences. A study utilizing fat-suppressed MRI techniques demonstrated that facet joint synovitis is a relatively common finding in patients with low back pain and may correlate with clinical symptoms [35]. These observations laid the foundation for more formalized scoring systems.

In 2016, Lee et al. introduced a novel MRI scoring method—the AS Activity of the Facet Joint (ASAFacet) score—to quantify inflammatory lesions in patients with ankylosing spondylitis (AS) [34]. This semiquantitative system assessed bone marrow edema (BME) adjacent to the facet joints and demonstrated good interobserver reliability. Importantly, facet joint inflammation measured by the ASAFacet score was significantly associated with systemic inflammatory markers, including ESR and CRP, supporting a relationship between MRI-detected facet-joint inflammation and systemic inflammatory activity in AS [34].

More recently, Acosta et al. proposed a comprehensive MRI-based grading system specifically targeting inflammatory changes of the lumbar facet joints [36]. This system evaluates three principal inflammatory features:

1. Facet joint effusion;
2. Bone marrow edema (BME) in adjacent osseous structures;
3. Periarticular soft tissue edema.

Facet-joint effusion is graded from 0 to 3, whereas BME and soft-tissue edema are assessed using 0–2 scales for intensity and extent. Intra-reader reliability ranged from κ = 0.42–0.81 and inter-reader reliability from κ = 0.37–0.54, supporting its potential use as a research tool while also highlighting limitations in inter-reader reproducibility [36].

The principal facet joint assessment approaches discussed in this review are summarized in Table 1. Notably, these methods were developed for different purposes: ASAFacet and the Acosta system assess inflammatory MRI abnormalities, whereas the Fujiwara and Weishaupt systems primarily characterize degenerative facet joint osteoarthritis [37,38].

These assessment approaches should not be regarded as interchangeable. ASAFacet was developed specifically to quantify inflammatory facet joint involvement in patients with established ankylosing spondylitis, whereas the Acosta system provides a more detailed feature-based assessment of inflammatory-appearing lumbar facet abnormalities but was developed in patients with symptomatic facet arthropathy rather than an axSpA diagnostic cohort. Conversely, the Fujiwara and Weishaupt classifications primarily assess degenerative facet joint osteoarthritis. None of these systems has been prospectively validated as a diagnostic instrument for distinguishing axSpA from mechanical or degenerative spinal disease, and none currently provides an established threshold for diagnosing axSpA or determining treatment eligibility.

A scoping review identified six studies describing MRI grading systems for lumbar facet-joint inflammatory changes. Commonly assessed features included hyperintense intra-articular signal or effusion, bone marrow edema, and peri-facet soft-tissue edema, although the specific features and grading approaches varied across studies [39]. Despite methodological variation, findings across studies suggest that these three components—joint effusion, adjacent bone marrow edema, and periarticular soft tissue changes—represent the core elements of inflammatory facet joint pathology.

However, no universally adopted international guideline (e.g., ASAS, OMERACT, or EULAR) currently exists for standardized scoring of facet joint inflammation. Variability in imaging protocols, anatomical levels assessed, grading thresholds, and reader expertise continues to limit comparability across studies. Moreover, routine radiology reports frequently omit documentation of inflammatory facet joint features, even when visible on MRI, contributing to underrecognition in clinical practice [26].

Inter-reader reliability remains an important methodological limitation in facet joint assessment. Reproducibility varies according to the lesion evaluated, the anatomical region, the imaging protocol, and reader experience. Facet-specific scoring systems have demonstrated variable agreement even when applied by experienced readers, highlighting the potential influence of lesion definition and image interpretation on grading. These observations support the use of explicit lesion definitions, standardized imaging protocols, atlas-based reference images, and reader training and calibration, particularly in research studies. Further validation is required before facet joint scoring systems can be incorporated reliably into routine clinical assessment.

Overall, MRI scoring systems for facet-joint inflammation are evolving toward semiquantitative, multidimensional frameworks that incorporate effusion, bone marrow edema, and soft tissue inflammatory changes. Although promising, further validation, harmonization, and integration into routine clinical reporting are required before these systems can be universally implemented in both research and everyday rheumatologic practice.

Although both anatomical regions are integral components of axial disease, important methodological differences exist between vertebral-based and facet-based scoring approaches. Vertebral MRI assessment in axSpA is well established and forms the backbone of imaging evaluation. Consensus definitions developed by the ASAS/OMERACT MRI group standardized the description of inflammatory spinal lesions, including anterior and posterior spondylitis, spondylodiscitis, costovertebral arthritis, and enthesitis [23]. A spinal MRI is considered positive for inflammation when anterior or posterior spondylitis is present in three or more vertebral sites [23].

Most vertebral scoring systems assess the following:

- Bone marrow edema (BME) at vertebral corners;
- Fatty deposition;
- Erosions;
- Syndesmophytes;
- Ankylosis.

These systems typically divide the spine into anatomical units (e.g., vertebral corners or discovertebral units), enabling systematic quantification of the inflammatory burden. Structural progression is frequently evaluated using radiographic scoring systems such as the modified Stoke Ankylosing Spondylitis Spine Score (mSASSS), which primarily targets anterior vertebral corners. Radiographic scoring systems such as the mSASSS provide standardized assessment of structural spinal damage, while incorporation of cervical facet-joint assessment may improve detection of structural damage and progression and strengthen associations with measures such as cervical rotation [28].

The strengths of vertebral scoring methodologies include standardized anatomical definitions, broad validation across studies, and incorporation into classification frameworks. However, they may underestimate disease burden by focusing predominantly on anterior vertebral corners and overlooking posterior elements, including facet joints.

Definitions of spinal MRI lesions in axSpA have continued to evolve. In 2022, the ASASMRI Working Group published updated definitions of inflammatory and structural spinal lesions based on consensus and multireader validation. The updated framework assesses lesions at the vertebral corners and endplates as well as the facet joints, transverse processes, and other lateral and posterior elements. The definitions include inflammatory bone marrow lesions and structural abnormalities such as fat lesions, erosions, bone formation, and ankylosis, providing a more standardized framework for the characterization of spinal involvement in axSpA [3].

Unlike vertebral scoring systems, which often use discovertebral units as reference structures, facet scoring relies on direct joint-based evaluation. The ASAFacet score introduced by Lee et al. quantified BME adjacent to the facet joints and demonstrated associations with systemic inflammatory markers, including ESR and CRP [34].

The grading system proposed by Acosta et al. assesses facet-joint effusion on a 0–3 scale and evaluates the intensity and extent of BME and peri-facet soft-tissue edema using 0–2 scales [36].

Several key methodological distinctions emerge:

1. Standardization and Validation. Vertebral assessment benefits from internationally standardized consensus definitions [23], whereas MRI-based facet-joint inflammatory scoring remains heterogeneous and incompletely standardized [39].
2. Anatomical Focus. Vertebral systems primarily assess anterior spinal elements and discovertebral units, while facet scoring evaluates posterior synovial joints. This distinction is clinically relevant, as inflammation of posterior spinal elements may be independently associated with restricted spinal mobility and functional impairment [29].
3. Inflammatory vs. Structural Emphasis. Vertebral methodologies frequently integrate both inflammatory and structural lesions, including fatty metaplasia and syndesmophytes. By contrast, facet scoring systems currently emphasize inflammatory features, with structural progression (e.g., ankylosis) less consistently quantified.
4. Correlation With Functional Outcomes. Evidence suggests that facet joint inflammation correlates strongly with reduced spinal mobility and worse functional indices (BASMI, BASFI) [29], potentially indicating that posterior joint involvement contributes disproportionately to mechanical restriction. Vertebral scoring systems also correlate with progression and structural damage, but may not fully capture the functional impact of posterior element inflammation.
5. Reporting in Clinical Practice. Facet joint inflammatory features are frequently underreported in routine lumbar spine MRI reports despite being identifiable on structured expert assessment [26]. This discrepancy highlights the gap between research-based assessment of facet inflammation and routine clinical reporting.

The development of composite radiographic scores, such as the Combined AS Spine Score (CASSS), which integrates cervical facet-joint evaluation with mSASSS, improved detection of structural damage and progression and showed a stronger correlation with cervical rotation than mSASSS alone [28]. However, these findings were derived predominantly from patients with established ankylosing spondylitis/radiographic axSpA and should not be extrapolated directly to non-radiographic axSpA. The prevalence, distribution, and prognostic significance of facet joint abnormalities in earlier and non-radiographic disease remain insufficiently characterized. Future methodological work should aim to harmonize vertebral and facet scoring frameworks within unified axial MRI assessment models. Standardized definitions, atlas-based calibration, and validation studies are needed to ensure reliability and facilitate integration into routine practice.

## 4. Degenerative Versus Inflammatory MRI Features

Differentiating inflammatory facet joint involvement related to axSpA from degenerative or mechanically induced facet arthropathy remains challenging. Structural findings such as joint-space narrowing, osteophyte formation, subchondral sclerosis, and cystic changes generally favor degenerative disease. However, fluid-sensitive abnormalities including facet joint effusion, bone marrow edema, and periarticular soft-tissue edema should not be considered specific for axSpA, because similar abnormalities may also occur in degenerative or mechanically stressed joints. Interpretation should therefore integrate lesion distribution, associated structural abnormalities, other spinal and sacroiliac MRI findings, age and mechanical loading factors, and the overall clinical context. At present, no individual facet joint MRI abnormality or combination of abnormalities has been prospectively validated as sufficiently specific to distinguish axSpA from degenerative facet disease in routine diagnostic practice. Several grading systems have been developed for the assessment of degenerative lumbar facet joint osteoarthritis. Fujiwara et al. developed an MRI-based grading system for facet joint osteoarthritis and demonstrated high agreement between MRI and CT assessment [37]. Weishaupt et al. evaluated lumbar facet joint osteoarthritis using a four-point grading scale and demonstrated moderate-to-good agreement between MRI and CT, although interobserver agreement differed between modalities [38]. These systems were developed to characterize degenerative facet joint osteoarthritis rather than inflammatory involvement in axSpA and should therefore be distinguished from MRI scoring approaches designed to assess inflammatory facet joint lesions.

## 5. Clinical Implications

Recognition of facet joint involvement on MRI may contribute to a more comprehensive characterization of spinal disease in patients with axSpA. Inflammatory abnormalities may include bone marrow edema adjacent to the facet joints, joint effusion, synovitis, and periarticular soft-tissue edema, whereas structural involvement may include erosive change and ankylosis. Previous studies have associated facet-joint inflammation with disease activity, restricted spinal mobility, and functional impairment, supporting the clinical relevance of posterior spinal involvement. Nevertheless, these associations should not be interpreted as evidence of independent diagnostic utility. The specificity of individual facet joint MRI abnormalities for axSpA has not been adequately established, particularly because similar findings may occur in degenerative or mechanically stressed joints. Likewise, the incremental value of facet joint assessment in patients with equivocal sacroiliac joint MRI findings or suspected non-radiographic axSpA remains uncertain. At present, facet joint MRI findings should therefore be interpreted within the overall clinical and imaging context rather than used in isolation to establish a diagnosis of axSpA or to determine eligibility for biologic therapy. Prospective diagnostic-accuracy and longitudinal studies are needed to determine whether facet joint assessment provides clinically meaningful information beyond established clinical, laboratory, and sacroiliac imaging parameters.

## 6. Limitations

This narrative review has several limitations. First, the literature search was non-systematic and restricted to PubMed; consequently, relevant studies indexed in other databases may not have been identified. Second, the available literature on facet joint involvement in axSpA is limited and methodologically heterogeneous, with substantial variation in study populations, MRI acquisition protocols, lesion definitions, scoring approaches, and reader experience. Much of the evidence regarding facet joint inflammation, ankylosis, structural progression, and clinical associations derives from patients with established or radiographic axial spondyloarthritis, historically classified as ankylosing spondylitis. Consequently, these findings cannot be assumed to apply directly to patients with non-radiographic axial spondyloarthritis or to individuals undergoing initial diagnostic evaluation. Furthermore, few studies have specifically evaluated the diagnostic accuracy or incremental diagnostic value of facet joint abnormalities, and appropriate degenerative or mechanical control groups have not been consistently included. The overlap between inflammatory-appearing facet abnormalities and changes related to degeneration or mechanical stress further limits specificity. Finally, the absence of a universally validated facet-specific scoring system and variability in inter-reader reliability currently limit implementation in routine clinical practice. Prospective, multicenter studies across the full axSpA spectrum, including non-radiographic disease and appropriate control populations, are required to establish the diagnostic, prognostic, and longitudinal value of facet joint imaging.

## 7. Conclusions

Magnetic resonance imaging (MRI) has fundamentally transformed the evaluation of axial spondyloarthritis by enabling early detection of inflammatory lesions before structural damage becomes apparent on conventional radiography. Although the sacroiliac joints remain central to imaging assessment, growing evidence supports the clinical relevance of facet joint inflammation as an additional manifestation of axial disease. MRI enables comprehensive evaluation of both active inflammatory changes and chronic structural lesions, while emerging semiquantitative scoring systems provide promising approaches for standardized assessment of the posterior spinal elements. However, facet joint MRI evaluation remains limited by the absence of internationally accepted definitions and validated scoring criteria. Continued refinement and standardization of MRI assessment will be essential to improve diagnostic confidence, better characterize the total inflammatory burden of axial spondyloarthritis, and support its integration into routine clinical practice.

## Figures and Tables

**Table 1:** Comparison of facet joint assessment and grading approaches relevant to axial spondyloarthritis and its differential diagnosis.

Assessment Approach	Modality	Principal Lesion(s) Assessed	Anatomical Region	Grading Approach	Validation/Reliability	Intended Use
ASAFacet (Lee et al.) [34]	MRI	Bone marrow edema adjacent to facet joints	C2-S1	Semiquantitative activity score across 23 discovertebral units	Good inter- and intra-observer reliability in 53 patients with established ankylosing spondylitis	Research assessment of inflammatory facet involvement in ankylosing spondylitis
Acosta et al. [36]	MRI	Facet joint effusion; bone marrow edema; periarticular soft-tissue edema	L3/4, L4/5, L5/S1	Feature-specific ordinal grading	Moderate inter-reader reliability; evaluated in 50 patients with symptomatic facet arthropathy	Standardized research assessment of inflammatory-appearing MRI features
Fujiwara et al. [37]	MRI with CT comparison	Degenerative facet joint osteoarthritis	Lumbar spine	Ordinal MRI grading of osteoarthritis severity	MRI assessment showed high agreement with CT in the original study	Characterization of degenerative lumbar facet osteoarthritis
Weishaupt et al. [38]	MRI and CT	Degenerative facet joint osteoarthritis	Lumbar spine	Four-point osteoarthritis scale	Moderate-to-good MRI-CT agreement; modality-dependent interobserver agreement	Characterization of degenerative lumbar facet osteoarthritis

## Data Availability

No new datasets were generated or analyzed in this review article.
